# Editorial

**DOI:** 10.1007/s40955-021-00181-8

**Published:** 2021-05-27

**Authors:** Elke Gruber, Thomas Jung

**Affiliations:** 1 Universität Graz, Graz, Österreich; 2 Deutsches Institut für Erwachsenenbildung – Leibniz-Zentrum für Lebenslanges Lernen, Bonn, Deutschland

Das erste Heft der *Zeitschrift für Weiterbildungsforschung* im Jahrgang 2021 entstand unter besonderen Bedingungen. Angekündigt wurde die Ausgabe im Herbst 2020 mit einem *Call for Papers*, der in aller Breite und Offenheit einlud, Forschungsergebnisse aus dem Feld der Weiterbildungsforschung einzureichen, ohne dass ein konkreter Schwerpunkt vorgegeben war. Dies war zu einem Zeitpunkt, als die zweite Welle der Covid-19-Pandemie anhob und Auswirkungen auf nahezu alle Bereiche des gesellschaftlichen Miteinanders spürbar wurden, die bis in die Gegenwart anhalten. Die Verhältnisse, unter denen gelebt, gearbeitet und geforscht wird, haben sich zum Teil erheblich verändert. In einer Zeit von Unvorhersehbarkeit und Unsicherheit ist die Wissenschaft umso mehr gefragt – wenngleich Antworten nicht immer so schnell wie erhofft gegeben werden können. Für Forschende in der Infektionsbiologie, Epidemiologie und Soziologie, aber auch für Bildungsforschende ermöglicht und erfordert es diese Zeit, neue Forschungsgegenstände in den Blick zu nehmen und innovative Forschungsfragen zu stellen. So war die Ankündigung für dieses Heft auch von der Hoffnung getragen, dass die Forschenden im Feld das Bedürfnis haben würden, ganz frische Forschungsergebnisse – mit und ohne Pandemie-Bezug – zu veröffentlichen. Und in der Tat wurden zehn Beiträge für diese Ausgabe angekündigt bzw. eingereicht, allerdings ohne auf Corona zu reagieren. Dies wäre vielleicht auch zu früh gewesen. Gleichwohl können wir ankündigen, dass noch in diesem Jahrgang ein Heft mit Beiträgen zu Pandemie-bezogener Forschung erscheinen wird.

Auch in einem weiteren Sinne ist diese Ausgabe unter neuen Konditionen entstanden. Die Zeitschrift ist mit Beginn des Jahres 2021 unter den Schirm des Projekts DEAL genommen worden. Das Deutsche Institut für Erwachsenenbildung (DIE) hatte im Dezember 2020 den DEAL-Vertrag mit der MPDL Service GmbH unterzeichnet, die als Vertragspartner die Umsetzung der Vereinbarungen zur Nationallizenz gegenüber dem Verlag Springer Nature verantwortet. Damit sind zum einen nahezu alle Zeitschriften des Verlags Springer Nature für die Mitarbeitenden in den an DEAL teilnehmenden Wissenschaftseinrichtungen in Deutschland frei zu lesen, zum anderen werden die Kosten für die *Publish-and-Read*-Lizenz bzw. die *Artikel Processing Charges *(APC) vom Konsortium bzw. den Instituten übernommen. APCs für Beiträge von Autorinnen und Autoren der *Zeitschrift für Weiterbildungsforschung* aus nicht an DEAL teilnehmenden Einrichtungen oder aus dem Ausland – ausgenommen sind hier Österreich und Schweiz, wo DEAL-kompatible Verträge abgeschlossen wurden – werden weiterhin vom DIE getragen. Für die Leserinnen und Leser ändert sich somit im Grunde nichts. Für die Autorinnen und Autoren gibt es ab diesem Jahr nur mehr eine zusätzliche Abfrage im Verlaufe des Einreichungsprozesses, bei der es darum geht, sich als DEAL-berechtigter Autor zu identifizieren. Weitere Informationen hierzu finden Sie auf der Webseite der Zeitschrift. Mit der Entscheidung, weiterhin als Open-Access-Zeitschrift unter dem Projekt DEAL zu firmieren, steht die Zeitschrift auch zukünftig für offene Forschung und Wissenschaftskommunikation. Nicht zuletzt hat auch die Pandemie gezeigt, wie wichtig der schnelle und kostenfreie Zugang zu Forschungsergebnissen im Open Access ist. Da das Projekt DEAL zunächst für drei Jahre befristet ist, wird über zukünftige Geschäftsmodelle und Finanzflüsse weiter nachzudenken sein. Wir vertrauen dabei in die kontinuierlich geführten Verhandlungen zwischen den nationalen Forschungsförderern und den nationalen bzw. internationalen Verlagen.

Im vorliegenden Heft können wir neben zwei Rezensionen von *Carola Iller* und *Reiner Treptow *zu aktuellen Büchern aus der Erwachsenenbildungsforschung nun drei Beiträge präsentieren. Die durch das Peer Review zu sichernden Qualitätsansprüche und das inhaltliche Profil der Zeitschrift ließen uns davon absehen, die anderen Einsendungen für eine Veröffentlichung zu berücksichtigen. Die drei Beiträge dieses Heftes ermöglichen einen höchst aufschlussreichen Einblick in drei unterschiedliche Schwerpunkte der Forschung zur Erwachsenen- und Weiterbildung.

In ihrem Beitrag zu „Ethnographie als Interaktionsprozess“ skizziert *Karola Cafantaris* Ansätze für eine ethnographische Erforschung hybrider Lernorte in der Erwachsenen- und Weiterbildung. Ausgehend von dem Befund, dass pädagogische Denk- und Handlungsstrukturen in nahezu alle Teile der Gesellschaft diffundieren und somit von einer „Allgegenwärtigkeit des Pädagogischen“ zu sprechen ist, verweist die Autorin darauf, dass die Ethnographie sich in den letzten Jahren als eine wichtige Strategie empirischer Forschung etabliert habe und auch innerhalb der qualitativen erziehungswissenschaftlichen Forschung zunehmend an Bedeutung gewinne. Auch für die Erwachsenenbildung kann die Autorin ethnographisch verortete Arbeiten ausmachen. Vor diesem Hintergrund gelingt es ihr, forschungsbezogene Merkmale hybrider Lernsettings herauszuarbeiten und Stärken der ethnographischen Forschungsmethode für die Erschließung hybrider Lernsettings zu erkunden. Dabei wird darauf fokussiert, wie die Forschenden das Untersuchungsfeld und die dabei entstehenden interaktionalen Prozesse in den Blick nehmen. In einem Fallbeispiel aus einem Geschichtsprojekt in der Erwachsenenbildung werden Kommunikationsformate und interaktionale Bezugsrahmen rekonstruiert. Mit ihrem Artikel leistet die Autorin einen ungemein wichtigen Beitrag zur Methodendiskussion in der Erwachsenen- und Weiterbildung, der zugleich darauf zielt, Möglichkeiten der Erforschung hybrider Lernsettings auszuloten.

Auch *Malte Ebner von Eschenbach* leistet mit seinen Überlegungen „Zur ‚culture continuée‘ Gaston Bachelards“ einen inspirierenden Beitrag zur Methodendiskussion in der Disziplin der Erwachsenen- und Weiterbildung. Ausgehend vom wissenschaftshistorischen Ansatz der Historischen Epistemologie, der für die Erziehungswissenschaften fruchtbar gemacht wird, richtet Ebner von Eschenbach den Blick auf die Produktion erziehungswissenschaftlichen Wissens und fokussiert dabei auf das Werk des bislang aus pädagogischer Perspektive wenig beachteten französischen Denkers Gaston Bachelards. Für die Re-Lektüre Bachelards nähert sich Ebner von Eschenbach dem Begründer der modernen französischen Epistemologie aus einer biografischen Perspektive, um sodann dessen akademisches und intellektuelles Profil herauszuarbeiten. Daran schließt sich eine vertiefende Auseinandersetzung mit Bachelards Untersuchung „Die Bildung des wissenschaftlichen Geistes“ aus dem Jahre 1938 an. Diese wird nicht nur vor dem Hintergrund des Konzepts eines „Erkenntnishindernisses“ gelesen, vielmehr wird herausgearbeitet, wie Bachelard darin das Verhältnis zwischen wissenschaftlichem Wissen und Alltagsdenken konzeptualisiert, das sich für ihn durch einen „epistemologischen Bruch“ auszeichnet. Mittels dieses Topos’ gelingt es Ebner von Eschenbach, Bachelards Konzept einer „ununterbrochenen Bildung“ vor einem erwachsenenpädagogischen Horizont Konturen zu verleihen. Mit diesen Überlegungen unterbreitet Ebner von Eschenbach ein Diskussionsangebot: dass es eine Funktion der Erwachsenenbildung sein könne, sich epistemischen Brüchen zuzuwenden und deren Reflexion zu befördern.

Im dritten Beitrag dieses Heftes untersuchen *Julia Koller, Jana Arbeiter und Michael Schemmann* die Akteurskonstellationen, Handlungskoordination und Leistungen in organisationalen Strukturen. Sie tun dies am Beispiel eines Feldes, das mit Blick auf die Finanzierung und Angebotsentwicklung als „fragil“ bezeichnet werden kann: das Feld der arbeitsorientierten Grundbildung. Dabei gehen sie von den theoretischen Ansätzen der Educational Governance sowie des Neo-Institutionalismus aus, um Fragen der Steuerung in dem benannten Feld zu analysieren. Die Autorinnen und der Autor untersuchen, wie soziale Ordnungen pädagogische Leistung ermöglichen. Ihr Fokus richtet sich dabei auf die Koordination von Handlungen in verschiedenen Akteurskonstellationen. So arbeiten sie heraus, welche institutionellen und organisationalen Strukturen in der arbeitsorientierten Grundbildung erkennbar sind und welche Formen der Handlungskoordination zwischen Akteuren welche pädagogischen Leistungen zustande kommen lassen. Empirische Befunde einer *multi case study*, die hier nachgezeichnet wird, weisen auf ein differenziertes und unterschiedlich dichtes Mehrebenensystem hin, in dem die Akteure über unterschiedliche Formen der Handlungskoordination Leistungen sichern und weiterentwickeln. Mit seinen abschließenden Hinweisen auf die Relevanz der Studie für die Praxis und die Bildungspolitik sowie mit dem Aufzeigen von Forschungsdesiderata leistet der Artikel einen innovativen Beitrag zur Debatte um Governance und Steuerung in der Erwachsenen- und Weiterbildung.

Wir freuen uns gemeinsam mit Ihnen, liebe Leserinnen und Leser, auf das kommende Heft, das, wie oben angekündigt, aktuelle Forschungsresultate zur Erwachsenen- und Weiterbildung präsentieren wird, die auf die Corona-Pandemie Bezug nehmen. Den *Call for Papers*, mit dem wir Sie zugleich ermutigen möchten, eigene Beiträge einzureichen, finden Sie demnächst in den einschlägigen Medien.

